# Soluble pattern recognition molecules: Guardians and regulators of homeostasis at airway mucosal surfaces

**DOI:** 10.1002/eji.201847811

**Published:** 2020-04-27

**Authors:** Ursula Smole, Bernhard Kratzer, Winfried F. Pickl

**Affiliations:** ^1^ Institute of Immunology Center for Pathophysiology Infectiology and Immunology Medical University of Vienna Vienna Austria

**Keywords:** Airway epithelial cells, Barrier tissues, Complement system, Innate defence, Soluble pattern recognition receptors

## Abstract

Maintenance of homeostasis at body barriers that are constantly challenged by microbes, toxins and potentially bioactive (macro)molecules requires complex, highly orchestrated mechanisms of protection. Recent discoveries in respiratory research have shed light on the unprecedented role of airway epithelial cells (AEC), which, besides immune cells homing to the lung, also significantly contribute to host defence by expressing membrane‐bound and soluble pattern recognition receptors (sPRR). Recent evidence suggests that distinct, evolutionary ancient, sPRR secreted by AEC might become activated by usually innocuous proteins, commonly referred to as allergens. We here provide a systematic overview on sPRR detectable in the mucus lining of AEC. Some of them become actively produced and secreted by AECs (like the pentraxins C‐reactive protein and pentraxin 3; the collectins mannose binding protein and surfactant proteins A and D; H‐ficolin; serum amyloid A; and the complement components C3 and C5). Others are elaborated by innate and adaptive immune cells such as monocytes/macrophages and T cells (like the pentraxins C‐reactive protein and pentraxin 3; L‐ficolin; serum amyloid A; and the complement components C3 and C5). Herein we discuss how sPRRs may contribute to homeostasis but sometimes also to overt disease (e.g. airway hyperreactivity and asthma) at the alveolar–air interface.

## Graphical abstract

Airway epithelial cells actively contribute to protection of the respiratory tract, by locally secreting soluble pattern recognition receptors (sPRR). The sPRR play critical roles in the recognition of conserved motifs and are linked to complement activation. This article reviews the current knowledge on the involvement of sPRRs in the pathogenesis of allergic airway diseases.

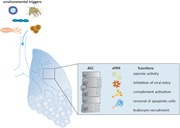

## Introduction

When resting, an average adult inhales about 11 000 L of ambient air per day. The inhaled air is neither sterile nor free of particles/macromolecules, and it potentially contains pathogens as well as allergens. Accordingly, the respiratory system has developed several safety mechanisms (e.g. mucus production by goblet cells, secretion of a plethora of antimicrobials, mucociliary transport by ciliated epithelial cells, coughing, etc.), which all together protect the body from harmful intruders at the alveolar–air interface [1]. Besides immune cells homing to this important organ, the cells lining the airways, that is airway epithelial cells, significantly contribute to host defence by expressing, amongst several other classes of antimicrobials, a multitude of pattern recognition receptors (PRR), which enables them to quickly respond to dangerous cues.

**Figure 1:**
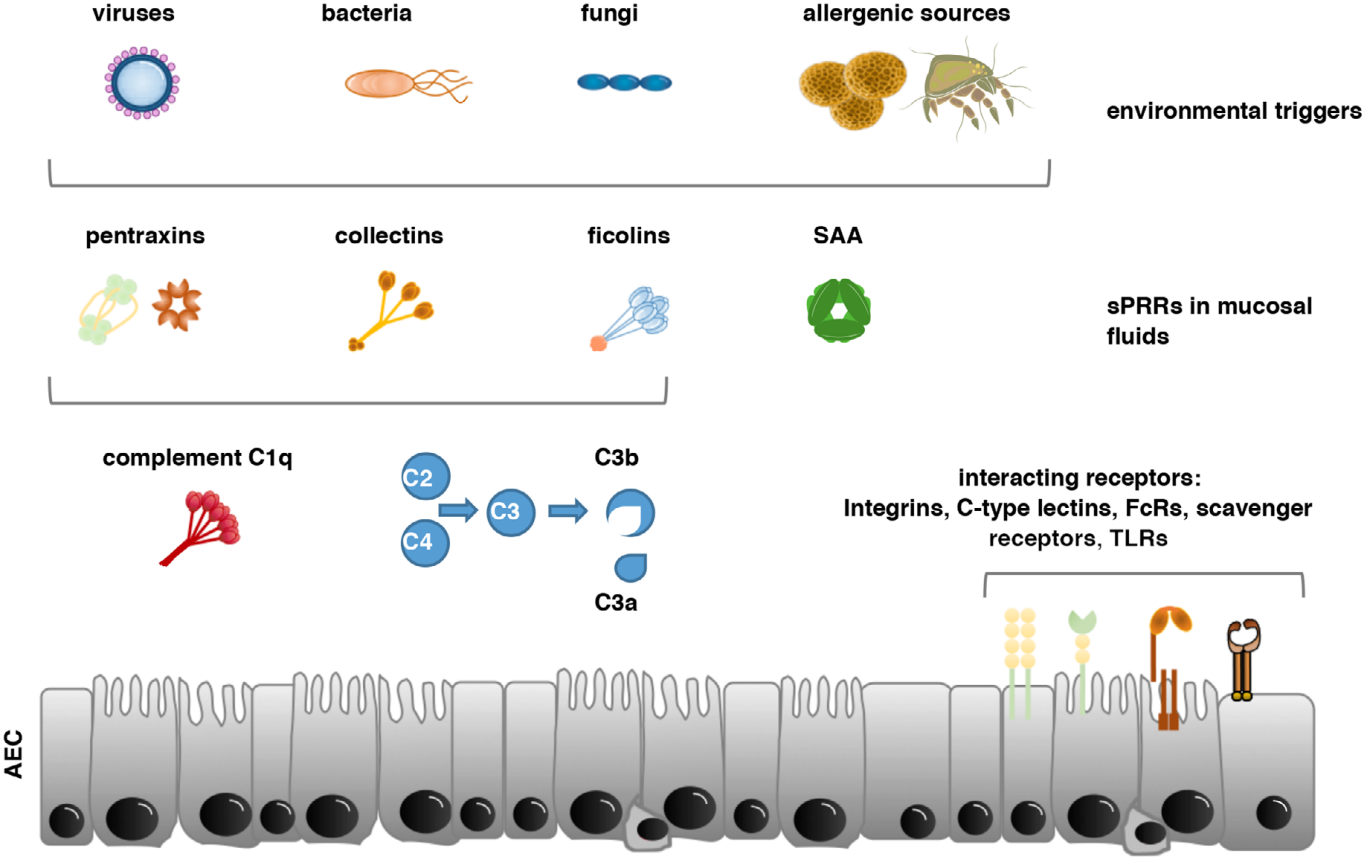
Humoral PRR within the mucosal lining of human airway epithelial cells. Shown are the different sPRRs within the mucus lining of AEC, their trigger factors and their primary mode of immune activation. The long pentraxin PTX3 has opsonic activity with a nonredundant protective role against selected pathogens, such as fungi, bacteria and viruses. Pentraxins are in addition complement activating proteins that lead to increased C3b levels and inflammatory signalling via C3a. The short pentraxins CRP and SAP are considered to bind to phosphorylcholine on pathogens and apoptotic cells, respectively. The collectin MBL is present primarily at mucosal surfaces including the airways and binds to a large array of pathogens, activating the lectin pathway of complement. Ficolins primarily function as opsonins, but can also activate the lectin pathway. SAA is a sPPR for Gram‐negative bacteria and blocks viral entry into cells. sPPRs also interact with and regulate the function of membrane bound PRRs such as Toll‐like receptors and C‐type lectin receptors but can also bind to integrins, Fcγ and scavenger receptors. Allergens might also have C3 convertase activity or might be recognized by C1q (in conjunction with specific antibodies). sPRRS might in addition function as an adjuvant and/or interact directly with environmental inhaled proteins to promote allergic sensitization.

In the literature, PRRs are classically described as cytoplasmic (nucleotide‐binding oligomerization domain‐like receptors/retinoic acid‐inducible gene‐like receptors) or cell surface expressed (i.e. C‐type lectin‐like receptors and Toll‐like receptors) receptors. Less well known is the role of soluble pattern recognition receptors (sPRRs), which represent a group of several other evolutionarily ancient but secreted molecules. Soluble PRRs are key players of the humoral arm of innate immunity [2, 3]. Under physiological conditions, these molecules are expressed constitutively at low levels in the liver by hepatocytes [4] and by a variety of immune cells, but their production rapidly increases in response to pathogens, initiating innate immune responses that modulate mucosal immunity in extracellular compartments such as the bronchial and alveolar airspace [2, 5, 6]. Notably, in the respiratory tract of mice and men, airway epithelial cells (AECs) are a major source of sPRRs (Fig. 1) and protect this barrier tissue from potential injuries (Table 1). Moreover, cells of the macrophage lineage [7] but also T cells, monocytes, dendritic cells (DCs) and granulocytes contribute to protection of the respiratory tract, by locally secreting sPPRs [8, 9] (Table 1). Soluble PRRs share the capacity to bind various microbial and environmental proteins and eliminate them through common mechanisms including agglutination, neutralization, opsonization followed by phagocytosis, with some of them having the capacity to activate complement [10, 11, 12]. As such, sPRRs possess antibody‐like features and might, in fact, represent their functional ancestors [13]. In addition, they also interact with and regulate the function of membrane‐bound PRRs, which cooperate at the level of the recognition of environmental patterns and the regulation of inflammation [12]. Accordingly, innate humoral immune responses at the respiratory mucosa are shaped by the presence of (i) sPRR with the ability to activate complement, that is, the pentraxins PTX3, CRP; the collectin mannose‐binding lectin (MBL); the ficolins ficolin‐2 and ‐3; (ii) sPRR unable to activate complement (SAA) and (iii) complement components themselves (C3).

**Table 1:** Cellular source of soluble pattern recognition receptors in the lung

sPRRs	Cellular source^a^	Evidence	Implication in allergic responses
***Pentraxins***			
Pentraxin 3 (PTX3)	Airway epithelial cells Airway smooth muscle cells Dendritic cells Macrophages Neutrophils	Secreted protein and mRNA [41, 66] Secreted protein and mRNA [41, 66] Secreted protein and mRNA [64] Secreted protein and mRNA [64] Store and secrete protein but no mRNA expression [65]	Increased BALF and serum levels in allergic asthma; levels correlate with disease severity [14, 21, 22, 41, 70].
C‐reactive protein (CRP)	Airway epithelial cells Alveolar macrophages	Secreted protein from cell lines and primary cells, mRNA [105, 106, 107] Secreted protein and mRNA [108]	Serum levels significantly elevated in asthmatics; correlate with reduced lung function and eosinophil numbers; associated with allergic sensitization and elevated risk of concomitant allergic airway inflammation [15, 34, 40, 98, 99, 103, 104].
Serum amyloid P component (SAP)	Liver		Increased sputum levels in asthmatics [14]
***Collectins***			
Mannose Binding Lectin (MBL)	Liver, foetal lung	Secreted protein and mRNA [116]	Directly binds allergen extracts [131]; increased plasma levels in asthmatics that correlate with eosinophil numbers [132, 133]
Surfactant protein A (SP‐A)	Alveolar type II cells club cells	Secreted protein and mRNA [136] Secreted protein and mRNA [136]	Both SP‐A and SP‐D can bind allergenic components including pollen, house dust mite and *A. fumigatus* [150, 151, 152]; SP‐D serum and BALF increased in allergic asthmatics [160, 161].
Surfactant protein D (SP‐D)	Alveolar type II cells club cells	Secreted protein and mRNA [137] Secreted protein and mRNA [138]	
***Ficolins***			
L ficolins (ficolin‐2)	Liver	Deficiency is associated with allergic and infectious diseases [179]	Direct involvement in allergic inflammation currently unknown.
H ficolins (ficolin‐3)	Airway epithelial cells	Secreted protein and mRNA [167]	
***Serum Amyloid A***			
Serum amyloid A (SAA)	Alveolar epithelial lining Alveolar macrophages monocytes	Secreted protein and mRNA [181] Secreted protein and mRNA [181] secreted protein and mRNA [182]	Elevated sputum SAA levels correlate with CRS and asthma prevalence and disease severity [23, 24, 212]
***Complement components***			
C3	Airway epithelial cells Dendritic cells Monocytes Neutrophils T cells	Secreted protein from cell lines and primary cells [226, 227, 237] Secreted protein and mRNA [241] Secreted protein and mRNA [241] Secreted protein and mRNA [241] Secreted protein and mRNA [241] intracellularly stored protein [8]	Allergens possess complement convertase activity and lead to C3a and C5a formation [243]; C3a levels are found upregulated in individuals with respiratory Allergies [250, 251]; positive correlation between C3 levels and asthma [252]
C5	Airway epithelial cells Dendritic cells T cells	Secreted protein from cell lines [228, 237] Secreted protein and mRNA [241] Secreted protein and mRNA [241] intracellularly stored protein [8]	

a Different cells are listed in alphabetical order.

Due to their dramatic systemic upregulation upon infection (i.e. by 500‐ to 1000‐fold), sPRRs have been termed ‘inflammation markers’, with some of them being extremely valuable in daily clinical practice (e.g. CRP, high‐sensitivity CRP and SAA) [14, 15, 16]. Airway mucosal inflammation in response to foreign antigens is an essential effector arm of innate host defence; however, the involvement of sPRRs in the regulation of the intensity and persistence of airway inflammation remains poorly understood. For instance, some of these sPRR, such as the collectin MBL [17] and the pentraxin PTX3, seem to inhibit, while others, like complement [18, 19], appear to aggravate infectious lung diseases triggered by deadly coronaviruses well known to induce severe acute respiratory syndrome and middle east respiratory syndrome [20].

Moreover, recent evidence suggests that sPRRs might also be causally involved in allergic airway inflammation [21, 22, 23, 24, 25]. In the following, we review the function of sPRRs present in the AEC mucus lining of mice and men and discuss their potential involvement in allergic inflammation in the lungs (Table 1).

## Pentraxins

**Figure 2:**
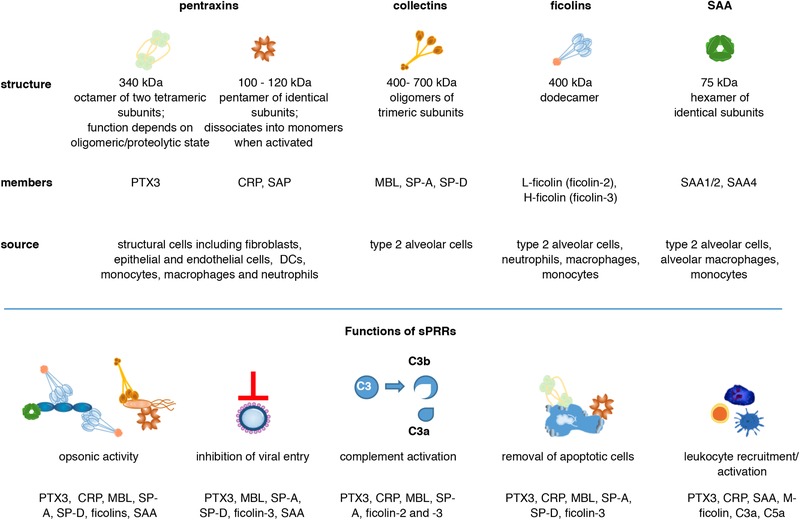
Protein structures, source and roles of sPRRs in the airways. Shown are the functional structures of pentraxins, collectins, ficolins and SAA proteins and their prominent family members. Local production of pentraxins in the lung could be detected in structural and myeloid cells as well as neutrophils. While the lung (type 2 alveolar cells) is the main synthesis site of the collectins SP‐A and SP‐D, MBL is primarily produced by the liver. Ficolins are produced in the liver by hepatocytes and in the lung by type 2 alveolar cells, as well as by neutrophils, macrophages and monocytes. SAA proteins, similar to pentraxins, were long thought to be liver‐derived serum factors but are also produced locally in the lung by type 2 alveolar cells, macrophages and monocytes. sPRRs share overlapping but also nonredundant roles in the first line of defence against microbial intruders through opsonic activity, inhibition of viral entry and the activation of the complement cascade. Some sPRRs play essential roles in the removal of apoptotic bodies, which is crucial for pulmonary immune tolerance and tissue homeostasis. In addition, several sPRRs are involved in leukocyte recruitment to the lung and local proinflammatory cytokine production.

Pentraxins belong to a superfamily of cyclic multimeric sPRRs (Fig. 2) that can be divided into short and long pentraxins based on their primary structure [11, 12, 13, 26, 27]. Prototypes of short pentraxins are the serum amyloid P component (SAP) and C‐reactive protein (CRP) [26] that constitute the main acute phase proteins in mouse and human, respectively [27]. Short pentraxins show a pentameric radial symmetry consisting of five or ten identical subunits of 25 kDa in size [13, 28, 29, 30]. While CRP and SAP are mainly produced systemically in the liver in response to inflammatory stimuli such as IL‐6, pentraxin 3 (PTX3), the most commonly known long pentraxin, is induced locally at sites of inflammation or cell damage where it plays a similar role as CRP and SAP in the circulation [11, 12, 31]. In contrast to CRP, measurement of systemic levels of PTX3 seems to be a much faster (peak levels reached at 7.5 h versus 24 h after hospital admission, respectively) and more reliable (faster regression to normal values) marker for acute myocardial infarction [32, 33]. Increasing evidence now also suggests that the expression of pentraxins is increased in asthmatic patients, contributing to allergic airway inflammation [14, 21, 22, 28, 34, 35, 36, 37, 38, 39, 40, 41]. Accordingly, pentraxins can be regarded as markers for (allergic) inflammation in the airways.

### Pentraxin 3 (PTX3)

#### PTX3: structure/function relationship

Thirty years ago, PTX3 was identified as a member of the pentraxin family and is now classified as the prototypic long pentraxin [42, 43]. PTX3 exists as a homo‐oligomer formed by eight identical, covalently linked subunits [44]. Protein oligomerization seems to be essential for PTX3 activity as other oligomeric forms of PTX3 have decreased functionality [45]. In addition, PTX3 function can be regulated by elastase and *Aspergillus fumigatus* proteases [46]. This indicates that PTX3 is capable to serve pleiotropic functions depending on its oligomeric and proteolytic state. As a sPRR, PTX3 has opsonic activity with a nonredundant protective role against selected pathogens, such as fungi, bacteria and viruses [2, 10, 13, 47]. PTX3 binds with high affinity to the outer membrane protein A from *Klebsiella pneumoniae* (KpOmpA) but does not interact with cellular receptors that bind this bacterial molecule. PTX3^−/−^ mice show reduced local inflammation in response to KpOmpA, indicating that PTX3 might amplify KpOmpA‐induced responses [48].

#### PTX3: epidemiologic data‐lung immunity

Most recently, PTX3 was shown to be a critical positive regulator for inflammatory responses during pneumococcal pneumonia [49]. While PTX3 deficiency renders mice more susceptible to pulmonary aspergillosis due to a defective recognition of conidia by antigen presenting cells and an inappropriate induction of an adaptive type 2 response [50], administration of exogenous PTX3 is able to rescue antifungal resistance early in infection [51]. Similarly, transgenic overexpression of PTX3 is responsible for resistance to viruses (e.g. influenza virus, cytomegalovirus), pathogenic (e.g. *Neisseria meningitidis*) and opportunistic (e.g. *Klebsiella pneumoniae* and *Pseudomonas aeruginosa*) bacteria, including preclinical models of bacterial sepsis induced by cecal ligation puncture [52, 53, 54]. PTX3 also takes part in extracellular matrix formation [45, 47] and interacts with recognition molecules of the classical and lectin complement pathway, Fcγ receptor, fibroblast growth factor 2 and P‐selectin [10, 50, 55, 56]. A number of clinical studies have pointed to the important role of PTX3 in lung and bone marrow transplant recipients [57, 58, 59, 60, 61, 62]. In fact, patients with elevated pretransplant PTX3 plasma levels are more likely to suffer from ischemia reperfusion injury leading to primary graft dysfunction after lung transplantation [57, 58, 59]. In contrast, genetic deficiency of PTX3 predisposes to invasive lung aspergillosis in patients who underwent bone marrow transplantation [61]. Elevated bronchoalveolar lavage fluid (BALF) PTX3 levels might be indicative of invasive aspergillosis in lung transplant patients [62] and represent a more reliable biomarker than determination of galactomannan, which gives a high rate of false positive results [60]. Moreover, serum PTX3 levels were found to be higher in patients with lung cancer as compared to cancer‐free heavy smokers [63]. In the airways, several innate immune cells have been found to produce PTX3 locally including macrophages, DCs [64] and neutrophils that store and secrete PTX3 as part of neutrophil extracellular traps [65], highlighting its critical role in the humoral arm of innate immunity. Besides infiltrating inflammatory cells, also structural cells, particularly bronchial and alveolar epithelial cells [66, 67] and smooth muscle cells, are main sites of PTX3 expression, which is absent in resting and activated T or B lymphocytes and NK cells [41, 68, 69].

#### PTX: role in allergic diseases

In human patients, PTX3 levels in BALF and sputum significantly correlate with disease severity [21, 22] and have been associated with allergic asthma in adults [14, 21, 22, 41, 70] and children [14]. Moreover, patients with allergic asthma show significantly higher PTX3 expression in airway smooth muscle cells as compared to healthy donors [41]. PTX3 enhances smooth muscle cell eotaxin‐1 release, which contributes to eosinophilic airway inflammation and airway remodelling [41]. In children, a positive correlation was identified between high sputum PTX3 levels and decreased pulmonary function. Similarly, significant positive correlations were identified between PTX3 sputum concentrations and eosinophil counts in sputum and blood as well as total serum immunoglobulin E (IgE) levels [22]. These findings have been confirmed recently in an independent study in a group of over 100 children with allergic asthma [71]. Notably, there is also a role for PTX3 in children with allergic rhinitis [37]. Children monosensitized to seasonal or perennial allergens and those with allergic rhinitis presented higher PTX3 plasma levels which also correlated with symptom severity [37]. Interestingly, the human PTX3 gene is located on chromosome 3q24–28 [42], a region associated with sensitization to Der p 1, a major allergen from house dust mites, and total serum IgE levels in humans [72]. A connection between PTX3 and type 2 immune responses was also established in an experimental model of ovalbumin‐induced allergic asthma in mice [21]. PTX3‐deficient mice exhibited enhanced type 2 inflammation in the lungs after ovalbumin sensitization and challenge with increased airway hyperresponsiveness, mucus plugging of the airways as well as BALF and lung eosinophilia [21]. The allergic phenotype was associated with a Th17‐dominant inflammatory response with an enhanced recruitment of granulocytes, particularly eosinophils and neutrophils, and increased IgE/IgG2a secretion [21]. Mechanistically, the Th17‐dominant allergic response in these mice was supported by the Th17‐polarizing cytokines IL‐6 and IL‐23 from DCs [21]. In summary, serum PTX3 levels might be a valuable marker to demonstrate the presence of low‐grade inflammation in asthma [71]. However, serum PTX3 levels seem to be inadequate as marker for asthma management, and it remains a matter of debate whether PTX3 might be directly involved in the etiology of allergic airway inflammation.

### C‐reactive protein

CRP has been named for its ability to bind and precipitate the ‘C’ polysaccharide contained in the pneumococcal cell wall in a Ca^2+^‐dependent manner [73]. Later on, this antigen has been identified as phosphocholine and related molecules on other microorganisms [74] but CRP also binds to phosphocholine exposed on the surface of damaged cells [75]. This involves CRP in the clearance of apoptotic cells [75]. Additional CRP ligands have been identified in recent years including nuclear antigens such as histones and chromatin as well as proteins from small nuclear RNP particles, fibronectin, laminin and polycations [76], which points to an important regulatory role of CRP in the development of autoimmunity [77].

CRP is an ideal marker for acute inflammation, showing a more than 1000‐fold increase in concentration under inflammatory conditions [75]. In recent years, more sensitive assays have been developed and high‐sensitivity CRP determination is considered as an indicator of low‐grade systemic inflammation that may be a useful predictor for the risk of atherosclerosis [78] and myocardial infarction [79]. It can also serve as an indicator of airway inflammation for many noncommunicable lung diseases including asthma [14, 34, 39, 40, 80].

#### CRP: structure/function relationship

The strongest activator for CRP transcription is interleukin‐6 [81], which requires the synergistic interaction of CCAAT/enhancer‐binding protein (C/EBP)‐beta and Rel p50 [82]. By contrast, interferon α significantly inhibits IL‐6‐induced CRP expression in hepatocytes [83], explaining why viral infections cause only modest elevations of CRP. Notably, the small molecule hypolipidemic drug Gemcabene not only lowers cholesterol but also CRP levels alone and synergistically with statins [84], and this is accomplished by interference with C/EBP‐delta and NF‐kB [85]. Once translated, monomeric CRP (mCRP; 21 kDa) assembles to pentamers in human plasma, which, in fact, might be in equilibrium with a decamer form under physiological conditions [86]. Mechanistically, CRP has homologous regions found in immunoglobins G (IgGs) and can therefore bind to FcγRI and FcγRIIa but also activate the complement cascade via C1q [87]. Moreover, an interesting dissociation mechanism has been described for pentameric CRP in the presence of bioactive lipids recently, suggesting that pentameric CRP which gives rise to mCRP [88, 89, 90, 91]. mCRP, formed on the surface of activated platelets, shows increased inflammatory properties and is rarely found in circulation, suggesting its predominant role in local inflammation that likely serves as a contributing factor atherosclerotic plaque formation [92]. However, mCRP also mitigates distinct immune reactions, for example, when bound to neutrophils via the FcγRIII, mCRP was shown to inhibit fMLP‐triggered chemotaxis [93], while aggregated CRP appeared to enhance reactive oxygen species production in response to aggregated IgG but not other stimuli, such as phorbol myristate acetate or opsonized zymosan [94]. Moreover, by recruiting factor H, mCRP also seems to be involved in blunting C3b activity, thereby contributing to the safe removal of opsonized bodily material [95].

#### CRP: epidemiologic data‐lung immunity

CRP is a well‐established biomarker of lung infection. In fact, CRP was first described in patients suffering from acute pneumonia due to *Streptococcus pneumoniae* infections [73]. Subsequently, increased CRP levels have been established as a systemic marker of reduced lung function also in chronic lung diseases such as cystic fibrosis [96] and chronic obstructive pulmonary disease [97]. The exact role of mCRP in the lung is still unknown.

#### CRP: role in allergic diseases

Serum CRP levels, as determined by high‐sensitivity CRP assays, have been found to be significantly elevated in asthma patients as compared to healthy controls [14, 34, 40, 98, 99], suggesting an association between plasma CRP levels and the severity of asthma [39, 100]. Indeed, elevated serum CRP levels have been correlated with decreased pulmonary function and increased sputum eosinophilic cationic protein levels and eosinophil numbers in steroid naïve asthma patients [34, 35, 39, 98, 99, 101, 102]. These studies were the first to indicate an association of systemic inflammation with airway inflammation. Similarly, studies in Danish school children as well as cohorts of U.S. children and adolescents reported that increased CRP levels were significantly associated with allergic sensitization and an elevated risk of concomitant allergic airway inflammation (rhinitis, asthma), however, independently of allergen type or clinical allergy symptoms [103, 104]. Yet, CRP cannot be used as a predictive biomarker in allergy as no associations were observed between CRP levels at 6 months of age with later development of allergic diseases [104]. Therefore, it was concluded that the low‐grade inflammation seen in children with allergic sensitization may be host intrinsic and part of a systemic inflammatory disorder rather than an isolated type 2 immune response or indirectly linked to sensitization through shared environmental risk factors (diet, microbiome) [104].

Apart from systemic alterations of serum CRP levels during lung diseases, there is clear evidence that CRP is also produced and secreted locally in the airways by epithelial cells such as nasal epithelial cells and the alveolar basal epithelial cell line A549 [105, 106, 107] and alveolar macrophages [108] Therefore, CRP may have direct and local effects on airway inflammation [109, 110] and contribute to bacterial clearance in the human respiratory tract [105].

With regard to the short pentraxin SAP, very little is known about its expression in allergic disease. Recently, Gao and coworkers found that sputum SAP levels in patients with eosinophilic asthma were significantly higher than in individuals suffering from noneosinophilic asthma or healthy controls [14].

## Collectins

Collectins are collagen containing C‐type lectins comprising MBL, surfactant proteins A and D as well as organ‐specific collectins.

### Mannose‐binding lectin

#### MBL: structure/function relationship

Human mannose (also mannan)‐binding lectin recognizes a large array of pathogens and activates the most ancient pathway of complement [111], very similar to C1q, which activates the classical pathway of complement [112]. MBL is composed of three identical 32 kDa polypeptide chains, which associate to a hexamer [113]. The individual polypeptide chains are composed of a carbohydrate binding and a neck domain, which is followed by a collagen domain. In circulation, MBL is complexed to the MBL‐associated serine proteases MASP‐1 and ‐2 [114, 115]. MBL is mainly expressed and produced in the liver [116].

#### MBL: epidemiologic data‐lung immunity

Studies in mouse and man have indicated that MBL is absent from the BALF of healthy individuals [117, 118], but can be clearly detected in the BALF of patients suffering from acute (pneumonia) [119] or chronic (protracted bacterial bronchitis) [120] respiratory diseases. Thus, detectable MBL levels in BALF can be regarded as a sign of inflammatory immune cell infiltration during infection [119].

With regard to its protective activity, MBL seems to be especially important during early childhood and in situations of immunosuppression. Some MBL variants fail to produce stable multimeric forms and are thus dysfunctional [116]. Indeed, MBL protects from infections and prolongs life expectancy in patients with congenital lung diseases such as cystic fibrosis, in which low MBL levels reduce life expectancy by 8 years [121]. The disease‐modifying role of MBL2 has been also correlated with the frequency of respiratory infection‐associated hospital admissions in chronic obstructive pulmonary disease patients [122], implicating a putative beneficial role for MBL replacement therapy in this disease entity [123]. Paradoxically, a recently performed long‐term follow‐up study showed that MBL‐deficient COPD patients had more polymorphic microbiota and less risk for infectious exacerbations when compared to patients with fully functional MBL. This was attributed to the fact that no oxidized and thus no macrophage‐inhibitory MBL could be formed in the collective of MBL‐deficient patients studied [124]. Hence, lung MBL can be regarded a double‐edged sword in situations of chronic inflammation, since oxidized forms of MBL might interfere with MBL‐oligomer formation and thus clearance of distinct microorganisms [125]

Apart from opsonizing pathogens and thus facilitating phagocytosis of microorganisms, MBL also largely contributes to the removal of apoptotic cells, especially within the lining of the lung alveoli, a process commonly referred to as efferocytosis [126]. Target cell bound MBL is recognized by calreticulin (the cC1qR), which is associated with CD91 (the α2 macroglobulin receptor), an endocytic receptor expressed on many cell types (hepatocytes, neurons, fibroblasts) including blood monocytes [127]. While the role of calreticulin for the uptake of MBL immune complexes is undisputed, some reports question a firm association of endocytosing calreticulin with CD91 [128]. This might indicate that other cell surface receptors are involved in calreticulin‐dependent endocytosis and thus require a redefinition of the clearance pathway for MBL bound. Intriguingly, the high frequency of MBL deficiency, amounting to 5% in the general population, represents a constant matter of debate, since only a selective advantage is able to establish such a high degree of ‘deficiency’ for a single protein playing otherwise an important role in humans. One could speculate that MBL deficiency potentially protects from autoimmunity, because in the absence of MBL the likelihood that the immune system becomes primed against constituents of apoptotic cell bodies or serum components is much lower [129]. Moreover, the absence of MBL might protect from intracellular parasitism/infections, since MBL might favour infection with mycobacteria (*Mycobacterium tuberculosis and Mycobacterium leprae*) [111]. However, also the exact opposite has been hypothesized, by showing that coincubation of T cells with MBL inhibits T cell activation, while the absence of functional MBL has been implicated to promote T cell driven autoimmune processes such as systemic lupus erythematosus and rheumatoid arthritis [130].

#### MBL: role in allergic diseases

MBL has been shown to bind to distinct allergen extracts and to activate complement via the lectin pathway, however, only in the presence of allergen‐specific IgG [131]. Moreover, significantly increased MBL plasma levels have been detected in children with asthma and in adults with asthma and associated allergic rhinitis [132], which correlated with peripheral blood eosinophil levels in children [133]. Similarly, patients suffering from *Aspergillus fumigatus*‐associated bronchopulmonary aspergillosis presented with elevated MBL serum levels [132], with the generated C3a and C5a fragments contributing to the bridging of innate and adaptive immune responses in asthma [134]. The possible involvement of MBL in the pathogenesis of airway hyperresponsiveness has been substantiated in preclinical studies in mMBL‐A knockout mice, which revealed a mitigated airway response upon challenge with *Aspergillus fumigatus* in the absence of mMBL‐A [135].

### Surfactant proteins‐A and ‐D

#### Surfactant proteins‐A and‐D: structure/function relationship

In the lungs, surfactant protein‐A (SP‐A) and surfactant protein‐D (SP‐D) are expressed and produced by alveolar type II cells and club cells of the distal bronchioles [136, 137, 138]. Similar to C1q, SP‐A proteins form a bouquet‐like structure [139]. Within the alveolar compartment, SP‐A is tightly associated with phospholipids. SP‐A opsonizes *Staphylococcus aureus* but also *Herpes simplex* particles for uptake by alveolar macrophages [140, 141]. In contrast, SP‐D obtains a cruciform structure, which links its trimeric subunits [142]. In addition, SP‐D also exists as a nonlipid bound free form, especially in alveoli. Apart from saccharides, SP‐D also binds to phosphatidyl‐inositol and glucosylceramide [143, 144, 145]. SP‐D plays an important role in the microbicidal activity within the alveolar space, since it opsonizes a number of Gram‐negative bacteria including *Escherichia coli*, activates the respiratory burst in alveolar macrophages [146] and leads to uptake of *E. coli* particles by DCs followed by T cell activation [147].

#### Surfactant proteins A and D: epidemiologic data‐lung immunity

Lung diseases, which increase alveolar‐capillary leakage, such as lung fibrosis or interstitial pneumonia, are associated with increased serum levels of SP‐A and SP‐D, while the respective BALF levels are reduced [148], making SP‐A and SP‐D markers for alveolar integrity. In addition, increases in especially of SP‐D levels have been observed to correlate with the degree of lung pneumonitis upon irradiation therapy [149].

#### Surfactant proteins A and D: role in allergic diseases

SP‐A has been shown to bind to a number of grass pollen grains in a Ca^2+^‐ and carbohydrate‐specific manner [150]. Moreover, both SP‐A and SP‐D bind to house dust mite [151] and *Aspergillus fumigatus* [152] antigens, binding to the latter inhibits the allergen's recognition by specific IgE antibodies and consequently blunts IgE‐dependent effector cell [153] and T cell [154] activation. In line with these findings, intranasal application of SP‐D in mice inhibited airway hypersensitivity induced by *Aspergillus fumigatus* [155, 156] and *Dermatophagoides pteronyssinus* [157], while challenge with *Aspergillus fumigatus* of SP‐A or SP‐D knockout mice promoted type 2 immunity involving hypereosinophilia and a high IL‐13/IFN‐γ‐ratio, which, again, could be abrogated by SP‐A and/or SP‐D application [158]. Of relevance, once IL‐13 becomes produced, it reduces SP‐D production by alveolar type II cells [159]. Irrespective of this regulatory mechanism, SP‐D serum [160] and BALF [161] levels were found to be increased in patients with allergic asthma.

## Ficolins

### Ficolins: structure/function relationship

Ficolins represent a class of opsonins that are characterized by the presence of a C‐terminal fibrinogen‐like and an N‐terminal collagen‐like domain (fi‐col‐ins) [162] that have distinct ligand specificities (GlcNAc GalNAc, sialic acid, d‐fucose). The fibrinogen‐like domain specifically binds to pathogen‐associated carbohydrates, while the collagen‐like domain signals ligand binding to the MBL‐associated serine proteases leading to their activation, eventually leading to the cleavage of C2 and C4 and the formation of C3 convertase activity [116]. Ficolins are lectins, which assemble to form oligomeric structures that look like a ‘bunch of flowers’ and have high, calcium‐dependent binding specificity for *N*‐acetyl‐glucosamine (GlcNAc), which is an important component of the cell wall of *Aspergillus fumigatus*. Although primarily functioning as opsonins, orchestrating the phagocytosis of fungi, ficolins also contribute to the activation of the lectin pathway by interacting and activating the MBL‐associated serine proteases [163] and trigger the production of inflammatory cytokines and nitric oxide by macrophages [164].

### Ficolins: Epidemiologic data‐lung immunity

Two serum soluble ficolins produced by hepatocytes exist, that is, L‐ficolin (ficolin‐2) [165] and H‐ficolin (ficolin‐3) [166]. Notably, the latter can be also produced and secreted by bronchial and alveolar type II epithelial cells [167]. Ficolin‐3 becomes increasingly produced by AEC upon systemic challenge with lipopolysaccharides (LPS) [168]. In addition, a membrane‐bound ficolin, M‐ficolin, for which binding to *Escherichia coli* has been described [169], is primarily expressed on monocytes [170]. While there is clear‐cut binding of ficolin‐3 to *Aspergillus fumigatus* conidia, its binding affinity is much lower than that of serum‐derived ficolin‐2. Moreover, low ficolin‐2 serum levels identify patients suffering from common variable immunodeficiency who are at risk to develop bronchiectases, a serious life‐shortening sequela of the disease [171]. Ficolin‐3 bound to sialylated glycans scavenges influenza A virus, which leads to the block of infectivity and hemagglutination activity of influenza A virus and its complement mediated destruction [172]. Ficolin‐3 deficiency due to mutations at position 1637 of the gene (1637delC) leads to immunodeficiency with recurrent lower respiratory infections, septicemia and warts [173], which can even lead to sudden death due to acute meningitis [174]. Interestingly, individuals heterozygous for the 1637delC mutation present with lower serum ficolin‐3 concentrations [175]. Moreover, ficolin‐3 has been shown to be responsible for the removal of late apoptotic cells [176]. This salient function might also be one of the reasons why ficolin‐3 is also a prominent target for autoantibodies (with DNA and other factors acting as adjuvant). Of note, anti‐ficolin‐3 antibodies are frequently (in one third) observed in systemic lupus erythematosus patients and currently have the strongest association of all autoantibodies with active lupus nephritis [177].

### Ficolins: Role in allergic diseases

The direct involvement of ficolins in allergic inflammation is currently unknown. However, a retrospective study suggests that low ficolin‐2 serum levels are associated with atopic disorders [178], which subsequently was confirmed in a prospective study indicating that ficolin‐2 deficiency is associated with allergic and infectious respiratory diseases [179]. The authors speculated that low ficolin levels might lead to a lack of protection from microorganisms potentially complicating (driving) allergic diseases [178].

## Serum amyloid A

Similar to the short pentraxin CRP, the serum amyloid A (SAA) proteins, have been classically viewed as highly inducible, liver‐derived factors in response to infection or trauma [180]. Today, baseline expression of SAA is well documented for many extrahepatic human tissues including the alveolar epithelial lining [181], alveolar macrophages [181] but also monocytes [182].

### SAA: structure/function relationship

SAA is a sPRR for Gram‐negative bacteria [183, 184] and blocks entry of viruses (e.g. hepatitis C virus) into cells [185]. More recently, SAA1 was found to directly bind LPS to form a complex that promotes LPS clearance by macrophages, which offers partial protection against LPS‐induced inflammation and acute lung injury [186]. In addition, SAA proteins can bind and transport retinol during bacterial infection [187], thereby restricting their optimal growth and expansion. Moreover, mice lacking *Saa3* express lower levels of retinoic acid receptors than their wild‐type littermates, which inhibits their ability to respond to retinoic acid and may lead to an incapacity to develop immune tolerance [188]. In humans, there are three SAA proteins that are encoded by the inducible genes, *SAA1* and *SAA2*, and the *SAA4* gene that is constitutively expressed [180, 189]. *SAA3*, which is a pseudogene in humans, encodes an additional inducible form of SAA in mice with high structural similarities to other SAA proteins [180, 189]. Locally produced, lipid‐free SAA1 can act through a number of potential SAA receptors [190] and has pleiotropic effects on the immune system including the recruitment of immune cells [191, 192, 193], epithelial wound repair [194] and proinflammatory cytokine production [185, 195, 196, 197, 198, 199, 200]. Considering the dramatic increase in SAA levels at sites of inflammation, the biological activities of SAA1 need to be tightly controlled, for example, by factors present in specific tissue microenvironments or by the ability of SAA1 to adopt different oligomeric states [187, 201, 202, 203, 204]. The structure of SAA, however, is different from chemokines and cytokines, and SAA seems to require ligand‐induced activation, which is not observed under physiological conditions [186, 205, 206]. For example, binding to plasma high‐density lipoprotein abrogates the cytokine‐like activities of SAA proteins [206].

### SAA: epidemiologic data‐lung immunity

*SAA3* knockout mice develop intrinsic airway hyperresponsiveness and show increased mortality to influenza A virus infection [188]. In mice, *Saa3* may therefore have key homeostatic functions in lung development and inflammation [188]. The sequence similarity of mouse Saa3 and human SAA1/2 suggests that their functions during inflammatory processes might be similar.

In the lungs, SAA1 has been established as a mediator of local effector Th17 responses [197, 207, 208, 209]. This is consistent with the role for SAAs in retinol shuttling that is required to induce Th17 responses in the presence of infection and inflammation [187]. SAA‐induced Th17 responses are associated with robust airway neutrophilia [207] and more severe asthma phenotypes that are less responsive to corticosteroids [210].

### SAA: role in allergic diseases

SAA is by far the most widely studied acute phase protein in the airways and has been repeatedly associated with chronic rhinosinusitis but also asthma symptoms [23, 24, 36, 186, 188, 207, 211, 212, 213, 214]. One such study analysed a fraction of the SARP‐3 study population (i.e. adult participants suffering from severe and nonsevere asthma), an NIH‐sponsored multisite cohort study conducted to investigate mechanisms of severe asthma. Notably, it was found that patients with severe asthma showed increased pruning of the pulmonary vasculature that was associated with decreased lung function, greater peripheral and sputum eosinophilia, and higher BAL SAA/lipoxin A_4_ ratio [87]. Moreover, there is a strong correlation between elevated blood as well as sputum SAA levels and asthma prevalence and/or allergic rhinitis and disease severity [23, 24, 212]. As described above, the ability of SAA1 to adopt different oligomeric states (monomers and oligomers) [202] or to transport retinols and related molecules [187, 188] might serve to scavenge and sequester essential vitamins (or even nutrients) to deter pathogen growth at mucosal surfaces [215] and may thus have important functional implications for promoting local inflammation. Considering that SAA is increased in allergic asthma [23, 24], it remains to be established whether SAA functions as an adjuvant and/or interacts directly with environmental airborne proteins to promote allergic sensitization. Preliminary evidence of the authors (Smole et al., in revision) suggests that a component within house dust mite extracts can lead to active forms of SAA which impact on AEC and the induction of type 2 immunity, thus contributing to the priming of exposed individuals for sensitization and allergic disease (Smole et al.). A better understanding of the diverse immune protective mechanisms of acute phase SAA at mucosal surfaces may help to develop novel therapies for infectious but also inflammatory diseases such as allergic asthma and rhinitis [186].

## Complement components

The complement system represents a central and very effective innate protection mechanism, which received its name from *Paul Ehrlich*, who intended to indicate that it enhances (complements) the activity of antibodies and phagocytes. It can be regarded as one of the centrepieces of humoral innate immunity and critically contributes to defence mechanisms in the airways. An emerging theme is, however, the recently discovered noncanonical functions of complement [216, 217], which indicate that this system has several important functions beyond being ‘the guardian of the extracellular space’ and being an ‘important player within the innate sensor network’. These functions include but are not restricted to its important contributions to neuronal cell and organ development and brain function [218], tissue repair, regeneration [219] and metabolic programming (also referred to as complosome) [220, 221, 222, 223], including the modulation of immune cell effector functions.

### Complement: structure/function relationship

The complement system encompasses more than 30 serum‐soluble and membrane‐expressed molecules [224, 225]. All three complement activation pathways (classic, nonclassic and lectin) eventually lead to C3‐ followed by C5‐convertase activity. Serine protease activity converts the evolutionary conserved C3 molecule, which is molecularly related to alpha‐2‐macroglobulin and highly abundant in human serum (1.2–1.3 g/L), into the small (10 kDa) and diffusible anaphylatoxin C3a and the larger opsonin fragment C3b [224, 225]. Similarly, C5 is converted into the small anaphylatoxin C5a (11 kDa) and the larger C5b fragment. The latter associates with C6 and C7, attaches to membranes (microbial or bodily) and completes the formation of the membrane attack complex by binding of C8 and C9 [223]. C3b‐dependent opsonization occurs upon conformational change of C3b and exposure of the hidden internal thioester bond which is highly reactive and covalently binds to nucleophils such as OH‐ or NH_2_‐groups in its immediate surroundings. The anaphylatoxins C3a and C5a are potent inflammatory mediators, which target a number of inflammatory and noninflammatory cell types.

### Complement: epidemiologic data‐lung immunity

The central components of complement, that is, circulating C3 and C5, are largely produced in the liver. However, it has been clearly demonstrated that apart from other nonliver cells, such as endothelial cells and immune cells (granulocytes, monocytes, DCs, T cells), also airway epithelial cells, such as BEAS‐2B cells, constitutively synthesize and release C3 under serum‐free conditions which can be significantly upregulated by IL‐1 or TNF‐α but not IFN‐γ [226, 227]. In addition, lung cancer cells substantially generate and release C5a, which is believed to create a milieu favourable for tumour cells and thus contributing to lung cancer progression [228]. Intratracheal instillation of C3a or peptides thereof induces respiratory distress, which is accompanied by C3‐dependent bronchial smooth muscle contraction in bronchioles and hemodynamic effects due to C3a‐induced platelet aggregation in pulmonary arteries [229, 230]. AEC (and airway smooth muscle cells) can sense complement, since both human and murine AEC express C3a‐ and C5a‐receptors under steady‐state conditions, which become upregulated in situations of endotoxinemia and allergen‐induced asthma [231]. Targeting of these receptors on AEC by intratracheal instillation of antibodies has been shown to reduce pulmonary oedema and lung injury [232, 233]. Apart from soluble complement components, AEC under steady‐state conditions also express transmembrane complement regulatory proteins such as Membrane cofactor protein (CD46), Decay accelerating factor (CD55) and protectin (CD59), with IFN‐γ able to significantly upregulate DAF levels [226]. It is therefore not surprising that complement proteins can also be detected in the BALF of healthy individuals [234] and that complement levels are increased upon exposure to bacterial danger signals such as LPS [235]. Some of these complement regulatory proteins might also contribute to the overall reduction of inflammation, such as CD46, which was shown to downregulate IL‐1β levels induced by H_2_O_2_‐activated AEC due to induction of autophagy and associated downregulation of inflammasome components like pro‐IL‐1β and NLRP3 [236]. Moreover, AEC were shown to scavenge (actively take up) C3 from their surroundings, very similar to CD4 T cells. In its intracellularly stored form, C3 is supposed to play a cytoprotective role by potentially influencing oxidative stress of AEC [237]. Production but also storage of C3 by AEC might have important implications also for end‐stage lung diseases such as cystic fibrosis and chronic obstructive pulmonary disease in which C3 stores in AEC are found to be increased. C3 is stored within the endoplasmic reticulum and late endosomes in its mature form, ready to become activated by cleavage. Other cell types known to not only synthesize but also store large amounts of C3 are neutrophils [238] and monocytes [239]. Moreover, T cells are not only activated by complement split products but also contain C3 and C5 intracellularly [240]. Whether T cells express and are able to activation‐dependently upregulate C3 and C5, or rather accumulate preformed C3 and C5 proteins present in large amounts in bodily fluids is a matter of intense discussion and might critically depended on APC‐T cell interaction [8, 241]. However, it has been clearly shown that T cells have complement convertase activity and can activate intracellular C3 by virtue of T cell‐expressed cathepsin L [9]. Secretion of such activated C3 (C3a) contributes to T cell‐mediated inflammation in target tissues including the lung. This also fits to the recent finding that intracellular pools of C3 become reduced upon T cell activation [8].

### Complement: role in allergic diseases

Early reports by Nagata and Glovsky have shown that different airborne allergens contain complement convertase activity and lead to C3a formation when incubated with serum [242]. Later on, these findings were corroborated by studies demonstrating that the serine protease from the house dust mite *Dermatophagoides farinae* generates the anaphylatoxins C3a and C5a [243], which already suggested a possible involvement of house dust proteases in the pathogenesis of allergic diseases [243]. Moreover, the mould component chitin, which is an important component of *Aspergillus fumigatus*, is a strong activator of the alternative complement pathway and thereby shapes the ensuing immune response upon inhalation [244].

Preclinical data confirmed the importance of anaphylatoxins in asthma pathogenesis. Along those lines, it was demonstrated that genetic deletion of C3aR on mouse AEC protects mice from lung dysfunction after allergen challenge [245]. Consistent with these findings, BALF of human patients with asthma [246, 247] but also respiratory distress syndrome [248] contain increased levels of C3a and C5a which might, at least in part, originate from AEC stores. Notably, the C3 pathway has been shown to be involved in the development of Th2 responses in allergic inflammation [245, 249], which is also reflected by the fact that C3a levels are found upregulated in individuals with respiratory allergies [250, 251] and asthma [252]. In addition, a positive correlation between C3 levels and asthma severity has been described [252]. Apart from lungs, also upper airway diseases, such as chronic rhinosinusitis, have been shown to be associated with increased local production of C3. Indeed, its neutralization has therapeutic effects in this difficult to treat condition [253].

In summary, the complement system and especially C3 is pivotal for the innate humoral protection of the airways. Humoral factors that are directly released by airway epithelial cells or immune cells associated with the epithelial cell lining diffuse into the mucus lining, and the majority of them eventually take advantage of the C3 convertase activity to signal the presence of danger.

## Summary

Mucosal surfaces represent very critical barrier tissues of our body that ensure immune homeostasis in response to microorganism that ranges from host defence to active tolerance and symbiosis. Consequently, and among other protection mechanisms, sPRR play an important role in local immune surveillance of mucosal surfaces and can quickly neutralize, opsonize or induce phagocytosis of potentially harmful intruders. Ligand binding by sPRR very effectively activates and amplifies canonical pathways of humoral immunity such as the complement system. The central component of it, that is C3, is also found in the airway mucosal lining. As such, sPRRs within the airway epithelial lining represent a very effective form of ‘forward defence’, especially against highly virulent and/or invasive strains of microorganisms characterized by strain‐specific sugar or lipid moieties (e.g. *Streptococcus pneumoniae*, *Aspergillus fumigatus*, etc.), and thereby prevent early and devastating epithelial damage to occur. Although seemingly redundant, sPRRs have largely diverging ligand specificities, several of them converge by activating the complement cascade. A large body of evidence suggests that sPRR might also sense the presence of usually innocuous proteins (allergens), setting the stage for allergic sensitization and subsequent allergic disease. Future research will focus on dissecting the underlying cellular and molecular complexity of potential sPRR–allergen interactions and highlight approaches of how to interfere with such undesired, allergen‐driven activation mechanism.

## Author contributions

U.S., B.K. and W.F.P. wrote the paper.

## Conflict of interest

Winfried F. Pickl holds stocks of Biomay AG and receives honoraria from Novartis and Roche. All other authors have no additional commercial or financial conflict of interest.

**Abbreviations**
AEC: airway epithelial cell
BALF: bronchoalveolar lavage fluid
CRP: C‐reactive protein
DCs: dendritic cells
MBL: mannose‐binding lectin
mCRP: momomeric C‐reactive protein
PTX: pentraxin
SAA: serum amyloid A
SAP: serum amyloid P
SP‐A: surfactant protein‐A
SP‐D: surfactant protein‐D
sPRR: soluble pattern recognition receptors
